# Assessing Disparities about Overweight and Obesity in Pakistani Youth Using Local and International Standards for Body Mass Index

**DOI:** 10.3390/jcm13102944

**Published:** 2024-05-16

**Authors:** Muhammad Asif, Hafiz Ahmad Iqrash Qureshi, Saba Mazhar Seyal, Muhammad Aslam, Muhammad Tauseef Sultan, Maysaa Elmahi Abd Elwahab, Piotr Matłosz, Justyna Wyszyńska

**Affiliations:** 1 Department of Statistics, Govt. Graduate College Qadir Pur Raan, Multan 60000, Pakistan; asifmalik722@gmail.com; 2 The Children Hospital and Institute of Child Health, Multan 60000, Pakistan; iqrash1010@gmail.com; 3 South City Hospital, District Headquarter (DHQ) Sadar, Multan 60000, Pakistan; usman-s86@yahoo.com; 4 Department of Statistics, Bahauddin Zakariya University, Multan 60800, Pakistan; aslamasadi@bzu.edu.pk; 5 Department of Human Nutrition, Bahauddin Zakariya University, Multan 60800, Pakistan; tauseefsultan@bzu.edu.pk; 6 Department of Mathematical Sciences, College of Science, Princess Nourah bint Abdulrahman University, Riyadh 11564, Saudi Arabia; meabdelwahab@pnu.edu.sa; 7 Institute of Physical Culture Sciences, Medical College of Rzeszow University, 35-959 Rzeszów, Poland; pmatlosz@ur.edu.pl; 8 Institute of Health Sciences, Medical College of Rzeszow University, 35-959 Rzeszów, Poland

**Keywords:** body mass index, CDC, IOTF, WHO, references, overweight, obesity

## Abstract

**Background/Objectives:** Obesity is currently considered a public health problem in both developed and developing countries. Gender- and age-specific body mass index (BMI) growth standards or references are particularly effective in monitoring the global obesity pandemic. This study aimed to report disparities in age-, gender- and ethnic-specific statistical estimates of overweight and obesity for 2–18 years aged Pakistani children and adolescents using the World Health Organization (WHO), the Center for Disease Control (CDC) 2000 references, the International Obesity Task Force (IOTF) and Pakistani references for BMI. **Methods**: The study used secondary data of 10,668 pediatric population, aged 2–18 years. Demographic information like age (years), gender, city and anthropometric examinations, i.e., height (cm) and weight (kg) were used in this study. The recommended age- and gender-specific BMI cut-offs of the WHO, CDC 2000 and the IOTF references were used to classify the children sampled as overweight and obese. For the Pakistani reference, overweight and obesity were defined as BMI-for-age ≥ 85th percentile and BMI-for-age ≥ 95th percentile, respectively. Cohen’s κ statistic was used to assess the agreement between the international references and local study population references in the classification of overweight/obesity. **Results**: The statistical estimates (%) of the participants for overweight and obesity varied according to the reference used: WHO (7.4% and 2.2%), CDC (4.9% and 2.1%), IOTF (5.2% and 2.0%) and Pakistan (8.8% and 6.0%), respectively; suggesting higher levels of overweight and obesity prevalence when local study references are used. The Kappa statistic shows a moderate to excellent agreement (κ ≥ 0.6) among three international references when classifying child overweight and obesity and poor agreement between local references and the WHO (0.45, 0.52), CDC (0.25, 0.50) and IOTF references (0.16, 0.31), for overweight and obesity, respectively. **Conclusions**: The results of the study showed a visible difference in the estimates of excess body weight after applying the WHO, CDC, IOTF and local BMI references to the study population. Based on the disparity results and poor agreement between international references and the local study reference, this study recommends using local BMI references in identifying children with overweight and obesity.

## 1. Introduction

Obesity has become a widespread public health concern globally, affecting both developed and developing nations. Studies have shown that excessive consumption of high-calorie foods, a lack of physical activity, and a sedentary lifestyle are prevalent among school-aged children, and these factors are the primary drivers of obesity [1,2]. The alarming increase in childhood obesity has been a major concern in recent years. According to worldwide data estimates, the prevalence of obesity in boys increased from 0.9% (0.5–13%) in 1975 to 7.8% (6.7–9.1%) in 2016, and in girls from 0.7% (0.4–1.2%) in 1975 to 5.6% (4.8–6.5%) in 2016 [3]. This public health issue has also been expended in developing countries including Pakistan. Data from several pieces of research in diverse contexts revealed that the prevalence of excess weight among Pakistani children has risen considerably, ranging between 5% and 20%, with rates higher in boys than in girls [4,5,6].

Epidemiological researchers regularly used the body mass index (BMI) as an internationally applicable indicator for assessing children’s growth and addressing the overweight and obesity problems. A child’s BMI is strongly linked to their body fat levels and future health risks, with a significant impact on long-term health outcomes during adolescence. Gender- and age-specific BMI growth standards or growth references are particularly effective for monitoring the global obesity pandemic. In 2000, the International Obesity Task Force (IOTF) used the data of children from six countries (*n* = 192,727): Brazil, Hong Kong, Singapore, Netherlands, Great Britain and the United States to establish a universally recognized definition for childhood overweight and obesity [7]. The World Health Organization (WHO) established a set of global growth standards for children from birth to five years old in 2006, using data from healthy pediatric populations from around the world [8]. The WHO introduced growth references for school-aged children and adolescents in 2007 [9], whereas the US Center for Disease Control (CDC) had already published its growth references in 2000 [10]. All three international obesity classification criteria are deemed valid, and one of them is usually employed in research or endorsed in local clinical practice guidelines. However, they raise the question of the comparability between them when applied to the same individual, and if they differ, which one is more precise. The selection of a reference is paramount, as it can have a significant impact on the accuracy of classification, and the use of an incorrect reference may lead to flawed conclusions and biased results.

Few studies [11,12,13,14] from developed countries have investigated the comparability of the WHO, IOTF and CDC references in evaluating the overweight and obesity status of children’s population. But, in developing countries, efforts to explore the comparability of each of these three references to the other in categorizing overweight and obesity are scarce. A contentious issue in the categorization of overweight and obesity in developing countries is whether international references are applicable, given that children in these regions tend to have lower BMI distributions and mature later than the reference population. This concern is evident, particularly in South Asian developing countries, including Pakistan, where growth characteristics in the pediatric population are different due to genetic and environmental variations from the populations used to develop international references [15,16,17,18]. A multi-ethnic anthropometric survey (MEAS) study formulated BMI-for-age growth references specifically for Pakistani children and adolescents aged 2–18 years [18], providing a tailored BMI-for-age reference for the Pakistani population. Therefore, the aim of this study is to report the age- and gender-specific statistical estimates of overweight and obesity for the 2 to 18-year-old pediatric population in Pakistan using three international references and the Pakistani reference for BMI. This study also compares the agreement between the WHO, CDC 2000, IOTF and the Pakistani references in the categorization of overweight/obesity.

## 2. Materials and Methods

We analyzed a dataset of 10,668 children aged 2–18 years collected by the MEAS survey, which has been thoroughly documented in a separate study [19,20,21]. The study, conducted through the MEAS survey, focused on a group of children from four major cities including four major cities, i.e., Multan (located in the south of Punjab), Lahore (located in central Punjab), Rawalpindi (located in North Punjab) and Islamabad (the capital city of Pakistan) [18]. Data from children aged 5 to 18 were gathered from various public and private schools (*n* = 68 schools) across the respective cities. The selection of children from each class was carried out through simple random sampling. For subjects under five years old, data were collected from various public locations, such as parks, shopping malls, and markets, using non-probability convenience sampling methods.

For this study, the demographic variables related to information like age (years), gender (boys/girls), residential city (Multan/Lahore/Rawalpindi/Islamabad) and anthropometric examinations, i.e., height (cm) and weight (kg) were used. Body weight and height (using a portable stadiometer SECA: SCA 217) were performed with the subject standing upright, following standard protocols. The complete procedure was discussed in detail elsewhere [19,20,21]. BMI was calculated using the formula: BMI = weight (in kilograms) divided by height (in meters) squared. The researchers ensured that the entire study adhered to the highest ethical standards.

### Statistical Analysis

The software “Statistical Package for Social Sciences (SPSS)” version 21.0 was used for data entry and performing all statistical analyses. Here is the revised text with corrected grammar: The normality of BMI data was assessed using the Kolmogorov–Smirnov test. Since the test revealed non-normality in the data, descriptive statistics were reported as the median and interquartile range (IQR). For comparisons between two groups, the Mann–Whitney U test was employed, while the Kruskal–Wallis test was utilized for comparisons involving more than two groups. Statistical estimates of overweight and obesity, categorized by age, gender, and residential city, were presented in frequency (*n*) along with corresponding percentages (%). For measuring overweight and obesity in children and adolescents, WHO growth standards for less than five years of age [8] and growth references for 5–19 years of age [9], CDC 2000 reference [10], IOTF growth references [7] and local BMI-for-age growth references developed by Asif et al. [18] were utilized. According to WHO growth standards or references, a subject having a BMI-for-age >1SD above the mean and >2SD above the mean was considered overweight and obese, respectively [8,9]. The USCDC 2000 growth reference system explained that a subject having BMI-for-age ≥ 85th percentile and BMI-for-age ≥ 95th percentile was considered to be overweight and obese, respectively [10]. The IOTF references provided percentile cut-offs corresponding to a BMI of 25.0 and 30.0 kg/m^2^ at 18 years of age for overweight and obesity, respectively. According to this criterion, a boy having BMI-for-age ≥90.5th percentile and ≥98.9th percentile was considered overweight and obese. While, the cut-offs for detecting overweight and obesity in girls were; ≥89.3th percentile and ≥98.6th percentile, respectively [7]. The Pakistani references were employed to classify overweight and obesity, with definitions set as BMI-for-age ≥ 85th percentile and BMI-for-age ≥ 95th percentile, respectively. While these percentile cut-offs mirror those outlined by the USCDC, they yield different threshold values for the local population. For instance, in the case of a 10-year-old boy, the 85th percentile results in a BMI cut-off value of 19.32, according to the USCDC, whereas it is 17.97, as reported in Asif et al. [18].

Cohen’s κ statistic was used to assess the agreement between the international references and our study population references in the classification of overweight and obesity. The values of κ < 0.6 and κ ≥ 0.90 were considered poor and excellent agreement, respectively [22]. A *p*-value < 0.05 was considered statistically significant. The study was approved by the Departmental Ethics Committee of Bahauddin Zakariya University, Multan, Pakistan (IRB# SOC/D/2715/19).

## 3. Results

The present study recruited 10,668 participants in the age range from 2 to 18 years {boys = 5539 (51.9%) and girls = 5129 (48.1%)}. Among those, the majority (26.61%) of the subjects were from Lahore city, followed by Rawalpindi or Islamabad city (16.53%) and Multan city (8.77%). The median BMI of the total subjects was 16.0 (14.40–18.29) kg/m^2^. Boys had significantly higher BMI values than those of girls (*p* < 0.001). The average BMI value of both boys and girls belonging to different ethnicities was also significantly different (*p* < 0.001) (Table 1).

Statistical estimates of overweight and obesity were substantially different across ages when three international and local references were applied. The results exhibited that 8.8% of the overall subjects (boys = 9.0% and girls = 8.5%) were overweight, and 6.0% (boys = 6.0% and girls = 5.9%) were obese by using the local study reference. By using the WHO, CDC and IOTF cut-offs, the overweight prevalence was 7.4% (boys = 6.9% and girls = 7.9%), 4.9% (boys = 4.5% and girls = 5.2%) and 5.2% (boys = 4.5% and girls = 5.8%), respectively and obesity was 2.2% (boys = 2.2% and girls = 2.2%), 2.1% (boys = 2.0% and girls = 2.3%) and 1.2% (boys = 1.0% and girls = 1.4%), respectively. We categorized our study population into two sub-age groups (2–10 years and 11–18 years) and three ethnicities (Lahore, Rawalpindi/Islamabad and Multan). Estimates of overweight and obesity prevalence based on local references were also higher among sub-age groups and ethnicities compared to the three international BMI cut-offs (Table 1 and Table 2).

Cohen’s kappa statistic findings for total participants revealed a poor agreement between local references and the WHO, CDC and IOTF references, i.e., κ = 0.45, 0.25, 0.16, respectively, for overweight and κ = 0.52, 0.50, 0.31, respectively, for obesity (Table 3).

## 4. Discussion

International references play a crucial role in facilitating comparisons between studies and countries, as well as in monitoring global trends of overweight and obesity. However, there is insufficient conclusive evidence to affirm their validity in developing countries [23]. It is widely observed that populations in developing countries often exhibit lower BMI reference values [15,18] compared to those outlined by the WHO, CDC, and other references from developed nations. This deviation may lead to overestimations in the prevalence of overweight and obesity if local references are not utilized. This observation was further supported by a recent study conducted in Pakistan by Qaisar and Karim [24], which revealed a significantly higher overall prevalence of overweight and obesity among girls when using local references compared to those from the WHO, CDC, and IOTF.

This study examines the prevalence of overweight and obesity among children and adolescents in Pakistan aged 2–18, providing gender- and ethnic-specific statistics. It also emphasizes the need to consider the suitability of global references for measuring overweight and obesity in Pakistani youth and their alignment with local cut-off points.

The MEAS data of 10,668 children and adolescents 2–18 years of age indicated that the estimates of overnutrition were found to be higher by the WHO references compared to the CDC and IOTF references (7.4% vs. 4.9% and 5.2% for overweight and 2.2% vs. 2.1% and 1.2% for obesity, respectively). The same trend was also observed among both boys (6.9% vs. 4.5% and 4.5% for overweight and 2.2% vs. 2.0% and 1.0% for obesity, respectively) and girls (7.9% vs. 5.2% and 5.8% for overweight and 2.2% vs. 2.3% and 1.4% for obesity, respectively). These findings were consistent with the earlier studies with the Algerian [25], Malaysian [26] and Saudi [27] child samples. A study in Lahore, Pakistan, found that 1860 schoolchildren had a higher prevalence of overweight and obesity when measured against the WHO reference compared to the IOTF and CDC references [28].

By using the local reference, the rates of overweight (8.8%) and obesity (6.0%) were higher compared to the aforementioned three different BMI classifications (i.e., WHO 2007, USCDC, and IOTF references). These results also support the previous study trend [24], showing that overweight and obesity rates among 8–16-year-old girls were higher when using local references compared to the results obtained by the three international references. The disparities in rates are largely attributed to variations in the criteria used to define and measure the threshold. The disparity in results may also be attributed to various related factors, including differences in the reference populations, such as the date of data collection, country of origin, study design, and genetic or environmental factors. The local study references were based on the cross-sectional data collected in 2016, while the IOTF references were constructed by using the dataset collected in six countries, mostly during the 1980s. In order to establish CDC references, two different datasets were collected in 1963–1965 and in 1976–1980 from the US children’s settings. The development of WHO growth references for school-aged children and adolescents was also based on pooling three data sets collected from the Health and Nutrition Examination Survey (HANES) Cycle I and Cycle II and Cycle III from the Health Examination Survey (HES). The inconsistency in statistical data on overweight and obesity highlights the need to consider whether universal international standards are suitable for all populations, as they may not accurately reflect the specific characteristics of certain groups.

In Pakistan, numerous published studies [4,5,6,29,30,31] were undertaken at a regional level, encompassing various age groups in which the criteria of WHO or CDC reference was used to deliver the estimates of overweight and obesity in children. Results in different settings indicated that the estimates for overweight vary between 8% to 19% and for obesity between 5% to 8%. The most recent cross-sectional study by Tanveer et al. [32] conducted in seven districts of central Punjab province with 3551 school children aged 9 to 17 years showed that 5.8% of the children were overweight and 5.4% were obese. Our study findings were also akin to the earlier study results.

Overall and across genders, the three international references showed a moderate agreement (0.6 ≤ κ < 0.8) among themselves when categorizing children as overweight. The agreement was moderate to excellent (κ ≥ 0.8) when examining child obesity, particularly between the WHO and CDC references. These results were also consistent with the Algerian and Malaysian study results [25,26] revealing that the agreement was excellent between the WHO and CDC for examining childhood obesity. In contrast, a comparison between three international references and the population’s own BMI reference showed a poor agreement (i.e., κ < 0.6), which is also parallel to earlier research results [24].

The study reveals that the choice of BMI reference can significantly impact estimates of overweight and obesity, ultimately affecting the strategies used by healthcare resources to address these issues and pediatricians’ decisions to provide clinical advice and management. It is not reasonable to rely solely on a single reference standard for all populations. Instead, we propose that each country develop its own local anthropometric reference based on its unique population data. These references must be thoroughly validated to ensure their accuracy in predicting and addressing potential health risks early on.

The treatment of various obesity-related diseases like cardio-metabolic and certain types of cancers is much more expensive, and timely assessment of abnormal weight status assists in reducing the economic burden. These statistical estimates of overweight and obesity will be helpful for pediatricians to combat abnormal weight status in childhood.

These can inform the development of targeted health policies to mitigate the negative consequences of being overweight and obese in the long term. Although we analyzed large and multi-ethnic sample data for representing statistical estimates of overweight and obesity, this study still has some limitations. Given the limitations of the MEAS dataset, which is comprised of a predominantly urban and affluent population, future research should strive to include diverse cohorts with varying ages and socio-economic backgrounds to broaden the generalizability of our findings. Moreover, a sedentary lifestyle and dietary intake behavior also have great influences on the BMI of children [1,2]. Therefore, comparative studies should also plan to include these significant factors. Despite the study’s limitations, we anticipate that our findings will contribute to the advancement of public health knowledge.

## 5. Conclusions

We observed a notable disparity in the estimates of overweight and obesity among Pakistani children and adolescents aged 2 to 18 when applying BMI references from the World Health Organization, Centers for Disease Control and Prevention, International Obesity Task Force, and local sources. The disparities uncovered and the limited agreement between international references and the local study reference suggest that this study advocates for the use of local BMI references to identify children with overweight and obesity. Population-specific BMI references undergo thorough validation to accurately assess health risks in the early stages, thereby aiding in the formulation of effective health policies to mitigate the long-term adverse effects of overweight and obesity.

## Figures and Tables

**Table 1:** Median (interquartile range (IQR)) body mass index and frequency (%) of overweight and obesity in boys by age and residential city area.

Age (Years)			Overweight				Obesity			
	*n*	Median (IQR)	*n* (%) ^a^	*n* (%) ^b^	*n* (%) ^c^	*n* (%) ^d^	*n* (%) ^a^	*n* (%) ^b^	*n* (%) ^c^	*n* (%) ^d^
2	19	12.49 (11.45–13.72)	2 (10.5)	1 (5.3)	1 (5.3)	1 (5.3)	1 (5.3)	--	--	--
3	100	14.34 (13.13–15.62)	15 (15.0)	3 (3.0)	3 (3.0)	4 (4.0)	8 (8.0)	6 (6.0)	6 (6.0)	4 (4.0)
4	208	14.42 (13.35–15.62)	18 (8.7)	11 (5.3)	6 (2.9)	5 (2.4)	5 (2.4)	5 (2.4)	6 (2.9)	3 (1.4)
5	240	14.61 (13.61–15.72)	16 (6.7)	18 (7.5)	14 (5.8)	8 (3.3)	19 (7.9)	19 (7.9)	21 (8.8)	17 (7.1)
6	292	14.61 (13.79–15.53)	14 (4.8)	14 (4.8)	14 (4.8)	15 (5.1)	16 (5.5)	16 (5.5)	16 (5.5)	11 (3.8)
7	279	14.62 (13.64–15.72)	20 (7.2)	23 (8.2)	18 (6.5)	23 (8.2)	21 (7.5)	21 (7.5)	20 (7.2)	7 (2.5)
8	273	14.81 (13.68–16.09)	12 (4.4)	14 (5.1)	9 (3.3)	13 (4.8)	18 (6.6)	17 (6.2)	16 (5.9)	8 (2.9)
9	247	15.14 (13.86–16.39)	17 (6.9)	20 (8.1)	17 (6.9)	19 (7.7)	16 (6.5)	11 (4.5)	8 (3.2)	3 (1.2)
10	420	15.38 (14.20–17.03)	47 (11.2)	34 (8.1)	25 (6.0)	25 (6.0)	19 (4.5)	12 (2.9)	8 (1.9)	--
11	439	15.68 (14.57–17.30)	40 (9.1)	40 (9.1)	27 (6.2)	25 (5.7)	27 (6.2)	10 (2.3)	6 (1.4)	--
12	675	16.02 (14.61–17.75)	54 (8.0)	64 (9.5)	43 (6.4)	35 (5.2)	45 (6.7)	6 (0.9)	2 (0.3)	--
13	593	16.42 (15.11–18.43)	49 (8.3)	58 (9.8)	28 (4.7)	27 (4.6)	42 (7.1)	--	--	--
14	563	17.33 (15.82–19.14)	60 (10.7)	37 (6.6)	25 (4.4)	26 (4.6)	30 (5.3)	--	--	--
15	546	17.85 (16.53–19.82)	52 (9.5)	32 (5.9)	18 (3.3)	19 (3.5)	35 (6.4)	--	--	--
16	381	18.59 (17.06–20.57)	40 (10.5)	13 (3.4)	2 (0.5)	5 (1.3)	22 (5.8)	--	--	--
17	169	19.38 (17.98–21.45)	31 (18.3)	--	--	--	7 (4.1)	--	--	--
18	95	19.57 (17.75–21.15)	13 (13.7)	--	--	--	4 (4.2)	--	--	--
Age-groups (years)										
2–10	2078	14.80 (13.66–16.07)	161 (7.7)	138 (6.6)	107 (5.1)	113 (5.4)	123 (5.9)	107 (5.1)	101 (4.9)	53 (2.6)
11–18	3461	17.12 (15.45–19.23)	339 (9.8)	244 (7.0)	143 (4.1)	137 (4.0)	212 (6.1)	16 (0.5)	8 (0.2)	--
02–18	5539	16.12 (14.58–18.26)	500 (9.0)	382 (6.9)	250 (4.5)	250 (4.5)	335 (6.0)	123 (2.2)	109 (2.0)	53 (1.0)
Residential city										
Lahore	2839	18.22 (14.57–18.36)	269 (9.5)	210 (7.4)	144 (5.1)	139 (4.9)	186 (6.6)	77 (2.7)	67 (2.4)	36 (1.3)
R. Pindi/Isl	1764	15.98 (14.48–18.03)	133 (7.5)	92 (5.2)	54 (3.1)	61 (3.5)	83 (4.7)	21 (1.2)	18 (1.0)	6 (0.3)
Multan	936	16.23 (14.79–18.30)	98 (10.5)	80 (8.5)	52 (5.6)	50 (5.3)	66 (7.1)	25 (2.7)	24 (2.6)	11 (1.2)

^a^: Overweight and obesity prevalence using local study reference; ^b^: Overweight and obesity prevalence using WHO reference; ^c^: Overweight and obesity prevalence using USCDC reference; ^d^: Overweight and obesity prevalence using IOTF reference; SD: Standard deviation; R. Pindi: Rawalpindi; Isl: Islamabad.

**Table 2:** Median (interquartile range (IQR)) body mass index and frequency (%) of overweight and obesity in girls by age and residential city area.

Age (Years)			Overweight				Obesity			
	*n*	Median (IQR)	*n* (%) ^a^	*n* (%) ^b^	*n* (%) ^c^	*n* (%) ^d^	*n* (%) ^a^	*n* (%) ^b^	*n* (%) ^c^	*n* (%) ^d^
2	43	13.00 (11.81–14.71)	3 (7.0)	2 (4.7)	--	--	1 (2.3)	1 (2.3)	1 (2.3)	1 (2.3)
3	170	14.33 (13.01–15.51)	10 (5.9)	5 (2.9)	3 (1.8)	4 (2.4)	10 (5.9)	8 (4.7)	8 (4.7)	6 (3.5)
4	313	14.57 (13.22–15.71)	31 (9.9)	17 (5.4)	13 (4.2)	12 (3.8)	20 (6.4)	14 (4.5)	18 (5.8)	13 (4.2)
5	381	14.42 (13.25–15.42)	23 (6.0)	26 (6.8)	21 (5.5)	20 (5.2)	29 (7.6)	19 (5.0)	25 (6.6)	17 (4.5)
6	405	14.35 (13.43–15.43)	25 (6.2)	28 (6.9)	23 (5.7)	23 (5.7)	12 (3.0)	9 (2.2)	11 (2.7)	8 (2.0)
7	381	14.72 (13.66–15.86)	27 (7.1)	31 (8.1)	25 (6.6)	29 (7.6)	19 (5.0)	15 (3.9)	15 (3.9)	9 (2.4)
8	376	14.88 (14.00–16.47)	31 (8.2)	41 (10.9)	31 (8.2)	36 (9.6)	25 (6.6)	18 (4.8)	15 (4.0)	10 (2.7)
9	336	15.33 (13.90–17.00)	36 (10.7)	41 (12.2)	28 (8.3)	34 (10.1)	18 (5.4)	11 (3.3)	11 (3.3)	5 (1.5)
10	459	15.83 (14.31–17.56)	44 (9.6)	45 (9.8)	23 (5.0)	28 (6.1)	17 (3.7)	9 (2.0)	8 (1.7)	4 (0.9)
11	325	16.56 (15.04–18.37)	21 (6.5)	32 (9.8)	28 (8.6)	35 (10.8)	30 (9.2)	11 (3.4)	7 (2.2)	--
12	436	16.89 (15.35–18.93)	42 (9.6)	53 (12.2)	32 (7.3)	35 (8.0)	31 (7.1)	--	--	--
13	460	17.58 (15.82–19.55)	51 (11.1)	44 (9.6)	24 (5.2)	24 (15.2)	26 (5.7)	--	--	--
14	341	18.26 (16.65–19.98)	--	--	--	--		--	--	--
15	257	18.67 (17.09–20.72)	--	--	--	--		--	--	--
16	191	18.92 (17.16–20.40)	--	--	--	--		--	--	--
17	129	18.97 (17.33–20.75)	--	--	--	--		--	--	--
18	126	19.26 (17.65–21.37)	--	--	--	--		--	--	--
Age-groups (years)										
2–10	2864	14.79 (13.65–16.15)	230 (8.0)	236 (8.2)	167 (5.8)	186 (6.5)	151 (5.3)	104 (3.6)	112 (3.9)	73 (2.5)
11–18	2265	17.86 (16.02–19.88)	208 (9.2)	167 (7.4)	102 (4.5)	114 (5.0)	153 (6.8)	11 (0.5)	7 (0.3)	--
02–18	5129	15.87 (14.27–18.31)	438 (8.5)	403 (7.9)	269 (5.2)	300 (5.8)	304 (5.9)	115 (2.2)	119 (2.3)	73 (1.4)
Residential city										
Lahore	2091	16.42 (14.47–18.75)	203 (9.7)	159 (7.6)	105 (5.0)	116 (5.5)	127 (6.1)	51 (2.4)	54 (2.6)	35 (1.7)
R. Pindi/Isl	1926	15.39 (14.05–17.57)	126 (6.5)	119 (6.2)	80 (4.2)	86 (4.5)	82 (4.3)	26 (1.3)	26 (1.3)	16 (0.8)
Multan	1112	16.11 (14.51–18.31)	109 (9.8)	125 (11.2)	84 (7.6)	98 (8.8)	95 (8.5)	38 (3.4)	39 (3.5)	22 (2.0)

^a^: Overweight and obesity prevalence using local study reference; ^b^: Overweight and obesity prevalence using WHO reference; ^c^: Overweight and obesity prevalence using USCDC reference; ^d^: Overweight and obesity prevalence using IOTF reference; SD: Standard deviation; R. Pindi: Rawalpindi; Isl: Islamabad.

**Table 3:** Agreement between international references in classifying overweight and obesity in a sample of Pakistani children.

Variable	Kappa Co-Efficient					
	Overweight			Obesity		
	IOTF	CDC	Pakistani Reference	IOTF	CDC	Pakistani Reference
Overall (*n* = 10,668)						
WHO	0.634	0.741	0.450	0.687	0.912	0.527
IOTF		0.808	0.160		0.707	0.316
CDC			0.258			0.503
Boys (*n* = 5539)						
WHO	0.595	0.722	0.775	0.597	0.912	0.522
IOTF		0.744	0.058		0.650	0.261
CDC			0.186			0.461
Girls (*n* = 5129)						
WHO	0.715	0.759	0.530	0.773	0.913	0.534
IOTF		0.864	0.266		0.756	0.373
CDC			0.336			0.548

IOTF: International Obesity Task Force, CDC: Centers for Disease Control and Prevention, WHO: World Health Organization.

## Data Availability

https://data.mendeley.com/datasets/sxgymx5xjm/1 (accessed on 13 May 2024).
